# Post-Varicella Arteriopathy as a Cause of Pediatric Arterial Ischemic Stroke: A Systematic Review and Case Report

**DOI:** 10.3390/brainsci15121333

**Published:** 2025-12-15

**Authors:** Martina Testaì, Silvia Marino, Giovanna Russo, Milena La Spina

**Affiliations:** 1 Postgraduate School of Pediatrics, University of Catania, 95100 Catania, Italy; 2 Unit of Pediatrics and Pediatric Emergency Care, University Hospital Policlinico “G. Rodolico-San Marco”, University of Catania, 95100 Catania, Italy; 3 Pediatric Hematology Oncology Unit, University Hospital Policlinico “G. Rodolico-San Marco”, University of Catania, 95100 Catania, Italy

**Keywords:** arterial ischemic stroke, post-varicella arteriopathy, varicella, vasculopathy, pediatric, MRI, antiviral treatment, MTHFR

## Abstract

Background/Objectives: Post-varicella arteriopathy (PVA) is a significant cause of pediatric arterial ischemic stroke (AIS) that typically involves previously healthy children within 12 months of primary varicella infection, mostly with a monophasic course. Diagnosis is based on clinical and imaging findings, and cerebrospinal fluid analysis may confirm it; treatment is empirical and heterogeneous. We describe a typical case of PVA and present a systematic review of its clinical, radiological, therapeutic, and outcome features. Methods: Following PRISMA 2020 and AMSTAR-2 guidelines, data on demographics, clinical presentation, imaging, laboratory confirmation, treatment, and outcomes were extracted across databases (PubMed, Embase, Scopus). Results: Forty-seven studies, encompassing 312 pediatric patients, were included. Mean age was 4 years with a median latency of 3.82 months from varicella to neurologic symptoms. Common presentation included hemiparesis, language impairment, and seizures. Imaging findings showed unilateral focal involvement of anterior circulation arteries, basal ganglia infarctions, and, rarely, bilateral or posterior circulation involvement. CSF VZV-DNA PCR and anti-VZV IgG were positive in 39% and 48% of tested patients. Treatment included intravenous acyclovir (34%), corticosteroids (20%), and low-dose aspirin (77%); two patients underwent acute reperfusion therapy (rt-PA or thrombectomy). Outcomes tended to be moderately favorable: 43% achieved full recovery, 45% had residual deficit, and 11% experienced recurrence. Prothrombotic state was reported, and it may influence disease severity. Conclusions: PVA is a rare distinct cause of pediatric stroke, with a generally favorable prognosis quoad vitam. Standardized guidelines and prospective studies are needed to establish evidence-based management. Clinicians should maintain a high suspicion for its diagnosis.

## 1. Introduction

Varicella (or chickenpox) is an infectious disease caused by the varicella-zoster virus (VZV), a neurotropic virus belonging to the Herpesviridae family. The World Health Organization (WHO) estimates that approximately 140 million cases of varicella occur globally each year, with 4.2 million serious complications requiring hospitalization and around 4200 related deaths [1,2]. Complications are rare in healthy children and may include bacterial superinfection of skin lesions, pneumonia, ocular involvement, and neurological manifestations such as encephalomyelitis, cerebellar ataxia, and VZV vasculopathy [3].

Several studies have shown an association between a history of varicella and the development of neurological symptoms and signs due to arterial ischemic stroke (AIS) or transient ischemic attack (TIA) within the following year in previously healthy children [4,5,6,7], after other major causes of stroke were ruled out.

According to the diagnostic criteria established by Lanthier et al. [8], this entity, known as “post-varicella arteriopathy” (PVA), is a unilateral stenosing arteriopathy affecting the supraclinoid internal carotid artery (ICA), the A1 or A2 segments of the anterior cerebral artery (ACA), or the M1 or M2 segments of the middle cerebral artery (MCA); moreover, brain imaging can also reveal infarcts within the vascular territory of the lenticulostriate branches (basal ganglia and internal capsule) [9]. PVA generally occurs within 12 months of VZV infection in immunocompetent children and is usually monophasic, often referred to as a transient cerebral arteriopathy (TCA) thanks to its stabilization or resolution within 6 months [8,10]. However, cases of progressive arteriopathy post-varicella AIS with delayed onset beyond 12 months [7], bilateral involvement [11], and atypical AIS locations [12,13,14] have also been reported. Diagnosis of PVA is supported by neurovascular imaging and may be confirmed by cerebrospinal fluid (CSF) analysis. The detection of amplifiable VZV-DNA, anti-VZV antibody (IgG) [15,16], or both can confirm the diagnosis of post-VZV stroke [17], although these markers are positive in only 30% of subjects, implying that a negative result does not rule out the diagnosis [12].

Treatment strategies include immediate antiviral therapy with intravenous acyclovir (10–15 mg/kg every 8 h) for 14–21 days and corticosteroids—which may be considered due to their anti-inflammatory effect—such as prednisone (1 mg/kg daily) for 5 days and antiplatelet therapy, such as acetylsalicylic acid, to prevent stroke recurrence [8,18,19,20].

We describe a typical case of transient post-varicella arteriopathy in a previously healthy child, which led to an arterial ischemic stroke with a favorable clinical resolution following combined antiviral, corticosteroid, and antiplatelet therapy. We also systematically reviewed the existing literature to describe PVA and its therapeutic approach.

## 2. Case Presentation

A 12-month-old male infant was referred for evaluation of suspected partial seizure-like episodes that began a few days prior to presentation, characterized by reduced movement of the left hemibody, predominantly occurring during awakening. Each episode lasted a few minutes, without altered consciousness, and was sometimes followed by irritability. The child was born at term following an uneventful pregnancy and delivery. The child’s psychomotor development was appropriate for his age. There were no reported infectious episodes in the previous weeks. Notably, the child had not received any vaccinations, and both the maternal and infant diets were strictly vegan, with complete exclusion of meat and all animal-derived products. These factors raised early concerns about possible micronutrient deficiencies and increased susceptibility to vaccine-preventable diseases. Neurological and general physical examinations were within normal limits.

**Figure 1:**
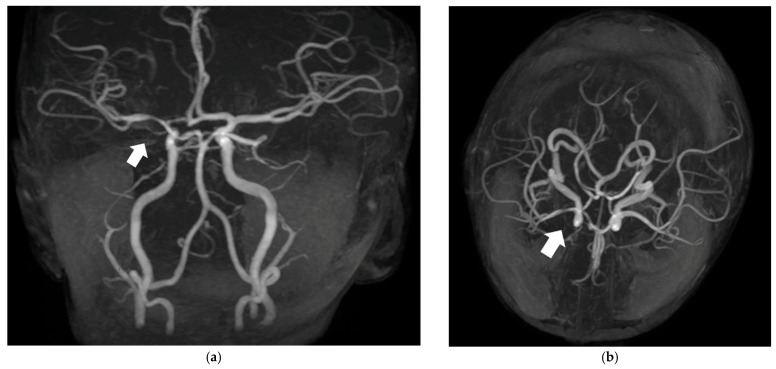
Brain magnetic resonance imaging (MRI). (**a**) Coronal angiographic sequence showing focal abnormalities, suggestive of vasculitis, of the proximal M1 segment of the right middle cerebral artery (arrow). (**b**) Axial angiographic acquisition showing focal involvement, suggestive of vasculitis, of M1 segment of the right middle cerebral artery (arrow).

During the diagnostic workup, focal abnormalities were identified on video-electroencephalogram (v-EEG) revealing focal epileptiform abnormalities. Anticonvulsant therapy with carbamazepine was started. Laboratory investigations revealed severe vitamin B12 deficiency and hyperhomocysteinemia, which were corrected with vitamin B12 (hydroxocobalamin 1.000 μg/day im) and folic acid supplementation (folic acid 100 μg/day os). Despite this, the episodes persisted. Cerebral MRI with angiographic sequences showed focal abnormalities suggestive of vasculitis involving the proximal M1 segment of the right middle cerebral artery, possibly due to transient embolic occlusion and subsequent inflammatory response (Figure 1). A critical part of his medical history emerged—namely that the patient had contracted varicella, a vaccine-preventable disease, five months prior to symptom onset, a detail not previously disclosed. At this stage, clinical and radiological findings became highly suggestive of post-varicella cerebral arteriopathy (PVCA). Cerebrospinal fluid analysis tested positive for VZV-DNA via PCR, confirming the diagnosis.

Treatment was started with intravenous acyclovir (10 mg/kg every 8 h for 21 days), oral prednisone (1 mg/kg/day for 5 days), and acetylsalicylic acid (3 mg/kg/day). First-level investigations for coagulation, autoimmune disorders, and metabolic disorders were unremarkable. A thrombophilia screen revealed heterozygosity for MTHFR gene polymorphism.

A follow-up MRI one month later demonstrated subacute evolution of the vascular lesions without new findings. Clinically, the patient showed complete resolution of focal symptoms.

## 3. Materials and Methods

### 3.1. Study Design

This systematic review was conducted in accordance with the 2020 PRISMA (Preferred Reporting Items for Systematic Reviews and Meta-Analyses) guidelines, and the checklist was completed (Supplementary Materials) [21]. The review protocol was registered in the Open Science Framework (registration ID 10.17605/OSF.IO/BN29J). According to the AMSTAR-2 assessment of the methodological quality of systematic reviews, all included reviews were rated as being of high quality [22]. The primary aim was to describe the clinical features, radiological findings, and treatment strategies of post-varicella arteriopathy (PVA) in the pediatric population.

### 3.2. Eligibility Criteria

We included studies that met the following criteria:

- Population: Pediatric patients (aged 0–18 years);
- Condition: Cerebral arteriopathy, vasculopathy, or arterial ischemic stroke occurring after varicella infection;
- Study types: Case reports, case series, and retrospective or prospective observational studies;
- Outcomes of interest: Clinical presentation, imaging findings, diagnostic approaches, treatments, and outcomes;
- Language: Articles published in English or with an English abstract available.

### 3.3. Exclusion Criteria

- Studies involving adults;
- Studies not clearly associating varicella infection with arteriopathy or stroke;
- Review articles, editorials, and conference abstracts (however, their references were screened for eligible primary studies);
- Non-English publications.

### 3.4. Search Strategy

A systematic search was conducted across databases (PubMed, Embase, Scopus). The search strategy used a combination of MeSH terms and free-text keywords, including “Varicella”, “Varicella zoster virus”, “Chickenpox”, “Post-varicella”, “Arteriopathy”, “Cerebral Arteriopathy”, “Focal cerebral arteriopathy”, “Vasculopathy”, “Stroke”, “Pediatrics”, “Children”, “Infant”, and “Adolescent”. Boolean operators (“AND”, “OR”) were used to refine the search.

Additional sources were identified through a manual review of reference lists from relevant articles.

### 3.5. Study Selection

All identified records were imported into Excel for screening. After removing duplicates, two reviewers independently screened the titles and abstracts for eligibility. Full texts of potentially relevant studies were then reviewed. Disagreements were resolved through consultation with a third reviewer. The selection process was documented using a PRISMA 2020 flow diagram.

### 3.6. Data Extraction

Data were extracted independently by two reviewers using a standardized form. The following information was collected:

- Study characteristics: Authors, year, country, study type;
- Patient demographics: Number of patients, age;
- Clinical data: Symptoms, timing of stroke after varicella;
- Imaging findings: MRI/MRA results;
- Diagnosis of PVA;
- Treatment modalities: Antiviral therapy, corticosteroids, antiplatelets/anticoagulants;
- Clinical outcomes.

Any discrepancies were resolved through discussion.

### 3.7. Data Synthesis

Due to the heterogeneity of study designs and outcome measures, a narrative synthesis was performed. No meta-analysis was conducted due to the descriptive nature of the included data.

### 3.8. Quality Appraisal

Two authors (S.M. and M.T.) independently assessed the risk of bias of the selected studies according to an 11-item instrument recommended by the Agency for Healthcare Research and Quality (AHRQ) for cross-sectional studies. For each item, we answered “yes”, “no”, or “unclear”, and studies receiving a score of seven or more stars were considered high quality. Disagreements were resolved by discussion with a third and fourth reviewer (G.R. and M.L.S.).

## 4. Results

### 4.1. Study Selection

**Figure 2:**
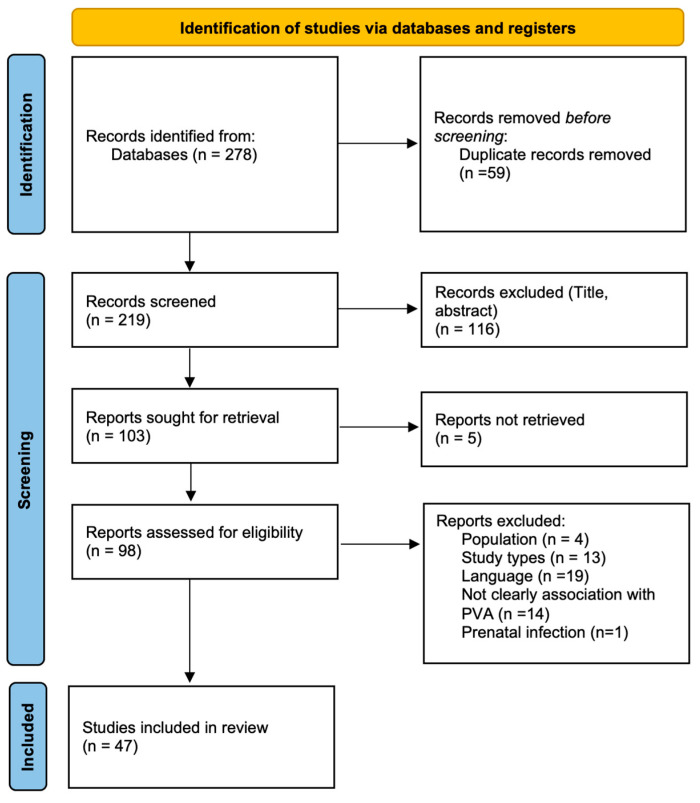
PRISMA 2020 flow diagram. Source: Page MJ et al. BMJ 2021;372:n71. doi:10.1136/bmj.n71 [21]. This work is licensed under CC BY 4.0.

The initial search across databases yielded 278 records. After removing 59 duplicates, 219 records were screened by title and abstract. Of these, 116 were excluded for not meeting eligibility criteria. Full-text articles were retrieved for 103 reports, but 5 could not be obtained. Of the remaining 98 articles, 47 studies met inclusion criteria and were included in the final synthesis. The study selection process is outlined in the PRISMA 2020 flow diagram (Figure 2).

### 4.2. Study Characteristics

The 47 included studies consisted primarily of single case reports and small case series published from 1992 to 2021. A minority were case-control and cohort studies. Most studies were from Europe, the UK, and the USA, with additional contributions from North America, Asia, and South America. The majority were retrospective.

### 4.3. Patient Demographics and Clinical Presentation

A total of 312 pediatric patients (age range: [1 month–18 years], median: [~4 years]) were identified with PVA following primary varicella infection. The mean latency between varicella infection and neurologic symptoms onset was approximately 3.82 months (mean), and onset typically occurred within 12 months. Nevertheless, a case of PVA was also described 12 months after reactivation of the vaccine-strain VZV in an immunocompromised patient. The most common presenting symptoms were as follows:

- Hemiparesis or hemiplegia (~79%);
- Language impairment (~34%);
- Seizures (~19%);
- Facial asymmetry or cranial nerves involvement (~17%);
- Choreiform movements (~17%);
- Altered consciousness (~8%).

Rare symptoms included headache, vomiting, hallucination, dystonia, nystagmus, dizziness, urinary incontinence, cardiorespiratory compromise, and ataxia (Table 1).

### 4.4. Imaging and Diagnosis

Magnetic resonance imaging (MRI) and magnetic resonance angiography (MRA) were the most used diagnostic modalities.

Common imaging findings, in accordance with the typical pattern of post-varicella arteriopathy, were:

- Unilateral and focal involvement of anterior circulation arteries, especially of MCA, followed by ACA and supraclinoid ICA;
- Ischemic infarcts in the basal ganglia and internal capsule regions in most patients;
- Posterior circulation involvement and multifocal lesions.

Notably, a small subset of cases (n = 5) exhibited atypical features, including bilateral arterial involvement, which is less commonly reported in the literature. Cerebrospinal fluid analysis was performed in 82 patients to confirm the VZV etiology:

- Positive VZV-DNA by PCR in 39% of tested patients;
- Positive anti-VZV IgG in 48% of tested patients.

The VZV antibody synthesis index, used to assess the intrathecal synthesis of VZV-specific IgG antibodies, was evaluated in 6 patients.

These confirmatory tests were not consistently performed across all studies (Table 1).

### 4.5. Treatment Modalities

Treatment strategies included the following:

- Antiviral therapy (IV acyclovir) in 34% of cases, for a duration of 14–21 days;
- Corticosteroids (prednisone or methylprednisolone) in 20% of cases;
- Antiplatelet therapy (ASA) in 77% of cases, often continued long-term.

Additional treatments included the following:

- Anticoagulants such as low molecular weight heparin (LMWH), warfarin, or dipyridamole or a combination therapy with antiplatelet and anticoagulant agents in selected cases;
- One case involved intravenous thrombolysis with recombinant tissue plasminogen activator (rt-PA);
- Another patient underwent a combined mechanical thrombectomy technique.

### 4.6. Outcomes

Clinical outcomes tended to be moderately favorable—particularly quoad vitam—with a substantial proportion of children experiencing residual neurological impairment. Key points:

- Complete recovery in 43% of children;
- Residual neurological deficits (e.g., mild motor deficits, seizures) in 45%;
- Recurrence of stroke was rare (reported in ~11% of cases);
- Mortality was reported in only 2 patients.

In most cases, follow-up neuroimaging revealed resolution or non-progression of arteriopathy reflecting the typically monophasic nature of the disease (Table 1).

## 5. Discussion

This systematic review collected data from 47 studies and 312 pediatric cases, reinforcing that post-varicella arteriopathy (PVA) is a significant cause of arterial ischemic stroke (AIS).

In particular, recent data from the prospective VIPS II (Vascular Effects of Infection in Pediatric Stroke) study further support the role of VZV in pediatric stroke, identifying subclinical reactivation as a contributing factor in a significant proportion of AIS cases, especially in children with focal cerebral arteriopathy [60].

PVA typically involves previously healthy children who experience an abrupt onset of neurological symptoms, most commonly hemiparesis, seizures, or language disorders, within 12 months of primary varicella infection [4,5,6,8,9,12,13,14,20,23,24,25,26,27,28,30,31,33,34,36,38,39,40,41,42,44,45,46,47,48,50,51,53,54,55,56,58,59]. Most cases follow a monophasic course, with unilateral stenosis of large vessels of the anterior circulation (MCA, ACA, or supraclinoid ICA) and infarcts in the basal ganglia region [5,6,7,8,9,13,20,23,24,25,26,27,28,30,31,33,34,36,38,39,40,41,42,44,45,46,47,48,49,50,51,52,53,54,55], according to the diagnostic criteria proposed by Lanthier et al. [8].

Aneurysm formation could be a rare complication of post-varicella arteriopathy. It is usually observed in immunocompromised patients [57]; however, one case reported a saccular aneurysm in an immunocompetent child, but it may have had a diathetic origin [35].

Notably, a small subset of patients exhibited atypical features, such as bilateral involvement [7,11,13,43,46,56] or posterior circulation stroke [14,35,51], suggesting that clinical spectrum of VZV vasculopathy may be broader than previously thought and emphasizing the need for extensive differential diagnosis and a high index of suspicion for this entity.

Both large and small vessels may be involved: large-vessel vasculopathy can result in extensive ischemic or hemorrhagic infarcts, whereas small-vessel disease may lead to focal ischemia, demyelination, or necrosis [18,61]. In addition, disruption of the internal elastic lamina in infected arteries may weaken the vessel wall, predisposing it to aneurysm formation, hemorrhage, or arterial dissection [18]. Histopathological studies demonstrate and confirm a direct viral-mediated mechanism. Evidence of varicella-zoster virus antigen within the arterial wall has been documented [52], and it is associated with multinucleated giant cells, Cowdry A inclusion bodies [12], and lymphocytic infiltrate [52].

The latency between varicella infection and neurological symptom onset averaged approximately 3.8 months, highlighting the importance of considering remote infectious history in children presenting with acute ischemic stroke. Although most cases were associated with primary VZV infection, at least one involved reactivation of the vaccine-strain VZV in an immunocompromised patient, raising important considerations for differential diagnosis in high-risk populations [43].

Nevertheless, a large population-based cohort study (including more than 360,000 children) showed no significant increase in the risk of arterial ischemic stroke within 12 months post-vaccination, providing strong reassurance about the vaccine’s safety [62]. Similarly, an editorial emphasized the importance of varicella vaccination based on a review of 70 pediatric post-VZV strokes, in which more than 50% of patients experienced permanent sequelae, arguing that nonfatal but nevertheless debilitating neurologic complications following VZV infection should guide immunization programs [63]. According to these observations, our reported case of post-varicella arteriopathy occurred in an unvaccinated child and emphasizes the crucial role of vaccination not only in preventing primary infection of exanthematous diseases—often perceived as benign, self-limiting illnesses—but also in reducing the burden of rare, devastating sequelae. In this context, the anti-vaccination attitude represents a serious public health concern, as it may expose children to preventable diseases with potentially life-threatening outcomes.

Laboratory-based confirmation of varicella involvement was variably performed through VZV-DNA PCR and/or anti-VZV IgG analysis in cerebrospinal fluid, yielding positive results in 39% and 48% of tested patients, respectively. These findings highlight the limited sensitivity of CSF testing and support previous literature conclusions that a negative result does not exclude PVA [12]. Very few studies assessed the VZV antibody index, which may be a more reliable indicator of intrathecal antibody synthesis [4,7,39]. Treatment approaches were largely empirical and heterogeneous, as no randomized controlled trials have specifically evaluated therapeutic protocols consisting of acyclovir, corticosteroids, or an antiplatelet agent, either alone or in combination. This aligns with expert recommendations and observational evidence supporting a multimodal approach targeting both viral replication and vascular inflammation [8,12,18] and using low-dose acetylsalicylic acid to reduce the risk of recurrence [10,64]. Bartolino et al., nonetheless, in a case series of four patients, highlight that only one patient received acyclovir and none received steroids (they were treated only with antiplatelet and/or anticoagulant drugs), but all showed clinical improvement, suggesting that further prospective studies are needed to assess the efficacy of steroids in the treatment of this condition [56]. In our review, more than three-quarters of patients received antiplatelet therapy, typically continued for several months.

Interestingly, two patients received acute reperfusion therapies—one with intravenous rt-PA [58] and one with mechanical thrombectomy [35]—suggesting that aggressive management may be considered in select patients.

However, prospective studies are needed to validate and guide standardized management.

Clinical outcomes tended to be moderately favorable quoad vitam: vascular lesions stabilized or resolved in most on follow-up imaging, and 43% of patients achieved complete clinical recovery. However, residual neurological deficits persisted in 45%, and stroke recurrence occurred in ~11%. Mortality was extremely rare, reported in only two studies [35,52]. Overall, these results suggest that PVA has a typically monophasic course with low mortality and recurrence rates, but a considerable proportion of patients experience long-term neurological impairment [4,5,6,7,8,20,24,25,29,32,36,38,41,42,44,46,47,48,49,50,51,55,56,58].

As we observed in our clinical case, underlying prothrombotic states may act as co-factors and should be investigated during the diagnostic phase. In particular, in our patient we hypothesize that a restrictive vegan diet may have induced vitamin deficiencies and consequent metabolic alterations, highlighting that acquired nutritional imbalance can also predispose patients to ischemic events. A case by Beleza et al. reported a patient with multiple transient ischemic attacks and PVA who was found to have a homozygous MTHFR C677T mutation, despite normal homocysteine levels, suggesting that the coexistence of PVA and a prothrombotic genotype may increase stroke risk and influence management decisions [5]. Similarly, Miravet et al. [13] and Ganesan V. [51], in their studies, identified several children with, respectively, heterozygous or homozygous MTHFR mutations and low levels of protein S, protein C, or antithrombin III among a cohort of patients with VZV-related AIS, further supporting the hypothesis that genetic predisposition may modulate disease severity and outcome.

However, this review has several limitations. The included literature consists primarily of case reports and small series, which may introduce publication bias and limit statistical power. In addition, clinical and radiological data were inconsistently reported across studies, and therapeutic regimens, as well as follow-up and outcome measures, were not standardized. These factors precluded the possibility of performing a quantitative synthesis or meta-analysis. Moreover, only studies published in English were included; relevant publications in other languages or in gray literature sources were not considered, potentially introducing language or publication bias. Finally, the absence of large prospective cohorts or randomized controlled trials makes it difficult to draw definitive conclusions about optimal treatment strategies.

## 6. Conclusions

PVA is a clinically distinct and typically monophasic cause of pediatric stroke, most often affecting previously healthy children within one year of varicella infection. Clinicians should maintain a high index of suspicion in patients with a recent history of VZV presenting with acute focal neurologic deficits, as diagnosis is primarily anamnestic and imaging-based; CSF analysis can provide additional support. We also underscore the importance of conducting a comprehensive thrombophilia workup, especially when the clinical presentation is atypical, there is stroke recurrence, or other vascular risk factors are present, because the presence of a prothrombotic alteration alone may not be sufficient to cause stroke, but in the context of endothelial injury from varicella-zoster virus, it may contribute to a multifactorial pathogenesis. Even if the prognosis quoad vitam is generally good, standardized guidelines for diagnosis, treatment, and follow-up are needed. Further prospective studies are essential to better define the long-term outcomes and to establish evidence-based management protocols. Furthermore, our case reinforces the crucial role of varicella vaccination in preventing primary infection and its severe complications, highlighting the importance of maintaining adequate immunization among the pediatric population.

## Figures and Tables

**Table 1:** Data collection table of the 47 studies included in our case report.

Study (Author, Year)	Country	Study Type	N° of Patients	Age Range/Mean Age	Time from Varicella to Stroke	Clinical Presentation	Imaging Findings (MRI/MRA)	Diagnosis of PVA	Treatment	Outcome
Vora SB et al., 2018 [23]	USA	Case report	1	11 mo	2–3 mo	Hemiparesis	Left MCA stenosis, stroke	Focal cerebral arteriopathy post-VZV CSF VZV DNA/IgG +	Acyclovir	Weakness improved, progressive arteriopathy at imaging
Rodrigues RM et al., 2022 [24]	Brazil	Case series	7	1.3–4 y/3.8 y	3.8 mo	Hemiparesis, aphasia, hemi-facial paralysis, focal seizures, dysarthria	(4) unilateral stenosis MCA or/and ICA and ACA, (3) infarction	Focal cerebral arteriopathy post-VZV, CSF VZV IgG + (3)	Acyclovir (2) Acyclovir + corticosteroids (1) ASA (7)	(3) no deficit, 2 mild hemiparesis, 1 epilepsy and hemiparesis No recurrence of AIS in 4 years
Helmuth IG et al., 2018 [6]	Denmark	Retrospective cohort	15	1–6 y/4 y	3 w–10 mo/4.6 mo (median)	Hemiparesis, facial nerve paresis, dysarthria, unilateral choreiform movements (1), seizures (2), headache or vomiting (8)	(12) unilateral stenosis of MCA or/and ICA, or/and ACA or basilar artery, (3) infarction BG	Focal cerebral arteriopathy post-VZV (12), CSF VZV DNA + (9) and IgG + (3)	Acyclovir (3), Acyclovir + corticosteroids (10) ASA (14)	(8) neurological sequelae No recurrence of AIS
Reis AF et al.,2013 [25]	Portugal	Case series and literature review	4	10 mo–4.5 y/2.33 y	1–10 mo/5.75 mo (median)	Hemiparesis, dysarthria	(3) right MCA stenosis, (1) stroke BG	Focal cerebral arteriopathy post-VZV or involvement of basal ganglia, CSF VZV DNA −	ASA, (1)LMWH + ASA	(3) residual dystonia, (1) hemiparesis. (2) complete resolution, (1) residual stenosis, (1) stable No recurrence of AIS
Bertamino M et al., 2021 [26]	Italy	Retrospective observational	22	1.7–10 y/4 y	2.8–8.6 mo/4.5 mo (median) 3 cases: 13–32.4 mo	Hemiparesis (73%), unilateral choreiform movements, language disorders, partial visual loss, strabismus and/or nystagmus, seizures, vomiting, altered state of consciousness	Focal stenosis of ICA, MCA, ACA; (7/22) only infarction of BG or brainstem Totally infarct of BG (18/22)	Focal cerebral arteriopathy post-VZV, CSF VZV DNA + (3/22) e IgG + (8/22)	Acyclovir (12/22) Acyclovir + corticosteroid (10/22) ASA (22/22) anticoagulant (11/22)	Motor deficits (12/22), cognitive impairment (8/22 Regression of stenosis (6/15), persistent narrowing (9/15) [4 improved, 3 stable, 2 worsened] (5) recurrent AIS
Lanthier et al., 2005 [8]	Canada	Retrospective cohort	23	1–10.4 y/4,4 y	4–47 w/17 w (median)	NR	Unilateral unifocal or multifocal stenosis MCA, ACA, ICA	Focal cerebral arteriopathy post-VZV	LMWH or/and ASA	Hemiparesis (13/23) hemidystonia (6/23) hemisensory deficit (3/23) speech problem (3/23), epilepsy (1/23) no deficit (9/23) AIS/TIA recurrence (8/23)
Science et al., 2014 [27]	Canada	Retrospective observational	10	2–11.5 y/4.5 y (mean)	2–26 w/4.25 w (median)	Hemiparesis, speech problem, facial weakness, blurred vision, hallucinations	Stenosis of MCA; infarct of BG	Focal cerebral arteriopathy post-VZV, CSF VZV DNA + (1/5)	Acyclovir (3)	hemiparesis (1/10), weakness of arm and short-term memory problems (1/10) increased tone of side (1/10), no deficit (7/10)
Thomas et al., 2013 [28]	UK	Case series	60	3.9 y (mean)	0–6 mo	NR	NR	VZV-associated AIS	NR	4-times increased stroke risk in 6 months post-VZV
Miravet et al., 2007 [3]	UK	Case series	24	2 m–6 y/2 y 9 m (mean)	1 w–12 m/4 m (median)	Hemiparesis, chorea, facial weakness, dysarthria, ataxia, seizure, decreased vision	Stenosis of MCA (1 bilateral), ACA, ICA, terminal internal carotid artery (1 bilateral), infarction of BG and subcortical white matter or cortical tissue	Focal cerebral arteriopathy post-VZV, CSF VZV DNA + (1/8) IgG + (14/14)	ASA or/and anticoagulants	Hemiparesis (18/24) behavioral problems (7/24) TIA (6/24) imaging of disease improved (11/24) resolution (1/24) increased or stable (12/24)
Darteyre S et al., 2012 [29]	France	Retrospective cohort	9 on 28	3.87 y (mean)	NR	NR	Unilateral and focal stenosis of MCA and terminal internal carotid artery 1 irregularity, 1 dissection	Focal cerebral arteriopathy post-VZV	ASA (1 no treatment)	no neurologic impairment (3/8), (2) cognitive impairment, (1) speech disorder, (5) motor impairment, (2) epilepsy No recurrence of AIS
Chiang et al., 2014 [30]	USA/Brazil	Case series	2 on 4	11 mo–3 y/1.96 y (mean)	15 d–1 y/0.52 y (median)	Hemiparesis, seizure	Posterior pontine subacute infarct, left BG infarct	Imaging post-VZV	Acyclovir	No follow-up
Askalan et al., 2001 [9]	Canada	Prospective cohort study	22	6 mo–10 y/4 y	1–11 mo/5.2 mo (median)	Hemiparesis, seizure	BG infarct, infarcts of anterior circulation, large vessel stenosis	Focal cerebral arteriopathy post-VZV or imaging of infarct post-VZV	LMWH (4/22) or/and coumadin (1/22) and/or ASA (13/22)	Neurological deficits mild (10), moderate (2), severe (3) no deficit (7) Recurrence AIS (10) with stenosis (9)
Braun K. P. J. et al., 2008 [31]	UK, France, Netherlands	Retrospective cohort study	32	0.3–16.3 y/4.8 y (for the TCA group, which includes PVA)	Within 12 months prior to stroke	Hemiparesis (1) no data for other	Unilateral focal stenosis MCA, ACA, ICA and basal ganglia infarcts	Focal cerebral arteriopathy post-VZV	ASA (58/7) of TCA (2) Acyclovir and ASA (10) anticoagulant	Recurrent neurological symptoms (13/74 with TCA) No patients in the progressive arteriopathy group had VZV
Ciccone S. et al., 2010 [32]	Italy	Case report	1	5 y	3 months after VZV reactivation (primary infection at 1 year old)	episodes of weakness of the left arm, left lower leg, walking difficulties, and dysarthria	Unilateral focal stenosis MCA ischemic lesion of BG	Focal cerebral arteriopathy post-VZV, CSF VZV DNA +	Acyclovir + LMWH + ASA	1 y later minimal reduction in motility of the left hand and in left leg coordination during sport activity, gliosis at RM
Dunkhase-Heinl et al., 2014 [33]	Denmark	Case Series	4	13–22 mo/17 mo (mean)	4 w–6 mo/3.4 mo (median)	Hemiparesis vomiting	Stenosis of MCA, ICA; infarct of BG	Focal cerebral arteriopathy post-VZV, CSF VZV DNA + (3/4) IgG + (2/4)	Acyclovir + corticosteroid (4/4), ASA (4/4)	Mild motor and cognitive deterioration (3) (3) normalization of stenosis, (1) residual stenosis, (1) stable
Bertamino M et al., 2021 [4]	Italy	Case report	1	6 y	7 mo	Mild motor difficulties, repetitive non-rhythmic movements of the right upper and inferior limbs	Infarct of the thalamus	Imaging post-VZV CSF VZV DNA − VZV antibodies synthesis index +	ASA first, acyclovir + corticosteroid later	No neurological sequelae 1 y later focal stenosis MCA and 6 months later persistent mild stenosis
Buompadre et al., 2009 [34]	Argentine	Retrospective and prospective case series	7 on 28	3 mo–6 y/3.6 y	15 d–6 mo/3 mo (median)	TIA (transient hemiparesis, arm weak- ness, gait disturbance) hemichorea, seizures	(3) stenosis of MCA, (1) occlusion of MCA, (7) infarct of GB and internal capsule	Imaging post-VZV	Not specified individually for VZV cases	(1) severe hemiparesis and dystonia, (2) mild hemiparesis, (1) dystonia, (3) no deficit. Normal vascular studies (5/7)
Fragata et al., 2021 [35]	Portugal	Retrospective case series	1 on 7	2 y	NR	Hemiparesis and somnolence	Occlusion of basilar artery, pons ischemia	Focal cerebral arteriopathy post-VZV CSF VZV DNA +	Thrombectomy	Fatal ischemia of the brainstem and cerebellum
Davico et al., 2018 [36]	Italy	Case series	2	3 y	4–6 mo/5 mo (median)	Hemichorea	Unilateral stenosis of ICA, MCA, ischemic lesions of BG	Focal cerebral arteriopathy post-VZV	ASA, corticosteroid, haloperidol	Clinical recovery, no recurrence of AIS
Hausler et al., 1998 [7]	Germany	Case series	4	4–16 y/9 y	6 w–4 y/1.8 y (median)	Transient impaired speech, vegetative symptoms, hemiparesis, aphasia, and disturbed consciousness	Unilateral focal stenosis MCA, bilateral occlusion of ICA, ischemic lesions of BG	Imaging post-VZV, CSF VZV DNA − VZV antibodies synthesis index + (3/4) VZV IgA serum (1/4)	Corticosteroid + LMWH + ASA (2/4)Acyclovir+ Corticosteroid + LMWH + ASA (1/4)	No deficit (2/4), cognitive impairment (2/4), hemiparesis (1/4) residual stenosis (2/4) No recurrence of AIS (3/4)
Moriuchi H et al., 2000 [37]	USA	Case report	1	12 y	9 months VZV reactivation	headache, weakness of left hemibody	Irregularity of MCA, edema of BG	Imaging post-VZV CSF VZV DNA +	NR	No neurologic deficit
Rougeot et al., 2006 [38]	France	Case report	1	2 y	1 mo	hemiplegia, somnolence, vomiting	Unilateral occlusion of MCA	Focal cerebral arteriopathy post-VZV, CSF VZV DNA − and IgG −	ASA	Expressive aphasia, moderate right upper limb weakness and neglect No recurrence of AIS
Kawatani et al., 2011 [39]	Japan	Case report	1	6 y	5 w	Hemiparesis, aphasia	Unilateral irregularity of MCA	Focal cerebral arteriopathy post-VZV, CSF VZV DNA − and IgG − antibodies synthesis index positive at follow-up	ASA	Mild dysfunction in fine-tuned movement of the right hand Stenosis of ACA e MCA, aneurism of ACA
Nguyên P. et al., 1994 [40]	France	Case series	1 of 6	2 y	3 w	Hemiparesis	Unilateral ischaemic zone of internal capsula	NR	NR	Evolution favorable
Driesen et al., 2015 [41]	Belgium	Case report	3	1.5–3 y/2.2 y	1.5–8 m/4.2 m (median)	Hemiparesis, speech disorder	Infarct of capsula interna, of BG and parietal region	Imaging post-VZV CSF VZV DNA + (2)	Acyclovir + corticosteroid + ASA (2) Acyclovir + Corticosteroid + LMWH followed by ASA	Complete recovery (3), no residual alteration at imaging (2)
Hayes B et al., 2007 [42]	Ireland	Case report	3	5–7 y/6 y	2 w–1 mo/0.8 mo (median)	Hemiparesis, gait abnormality, dystonia, hemi-facial dropping, dysarthria	Unilateral stenosis of MCA, occlusion of ICA, infarct of BG	Imaging post-VZV CSF VZV DNA and IgG + (1)	Acyclovir + corticosteroid + ASA (1) Acyclovir + corticosteroid	Epilepsy, moderate deficit of arm, mild of lower limb, progression of stenosis (1) no progression (2) No recurrence of AIS (2)
Sabry et al., 2014 [43]	USA	Case report	1	6 y	1 y after reactivation (only VZV vaccination)	Hemiparesis, paresthesias, dizziness, urinary incontinence	Multifocal stenosis of ACA, anterior communicating artery and bilateral stenosis of MCA, supraclinoid ICA	Imaging post-VZV, CSF VZV DNA + compatible with varicella vaccine strain	Acyclovir + corticosteroid + ASA	No neurologic residual
Bodensteiner JB et al., 1992 [44]	USA	Case series	5	3–7 y/5.8 y	3–8 w/5.4 w (median)	Hemiparesis, seizure, headache, lethargy	Unilateral focal stenosis of MCA (1/5) cortical stroke (4/5), deep stroke (2/5)	Imaging post-VZV	Corticosteroid + ASA (1) corticosteroid (1), dipyridamole (1)	No neurologic residual, no recurrence of AIS
Yilmaz K et al., 1998 [45]	Turkey	Case report	1	18 mo	10 d	Hemiplegia, gait disturbance, seizure	Infarction of BG and internal capsule	Imaging post-VZV	NR	Mild hemiparesis
Magagnini MC et al., 2015 [20]	Italy	Case report	1	5 y	1 mo	Hemiplegia, speech impairment	Infarction of BG	Imaging post-VZV	Acyclovir	No neurologic residual, no recurrence of AIS
Morino et al., 2009 [46]	Japan	Case report	1	2 y	9 d	Hemiparesis	Bilateral stenosis of MCA, ACA and ICA (supraclinoid)	Imaging post-VZV	ASA	Improvement of neurologic condition, no recurrence of AIS
Singhal AB et al., 2001 [47]	USA	Case report	1	14 y	4 mo	Hemiparesis, aphasia, diplopia, monocular blindness	Unilateral stenosis of MCA, ICA; infarct of BG	Imaging post-VZV CSF VZV IgG +	Acyclovir + Corticosteroid + ASA	No neurologic residual, resolution of stenosis, no recurrence of AIS
Danchaivijitr N et al., 2006 [48]	UK	Case report	1	7 mo	2 mo	Somnolence, apnea, bradycardia at onset, after hemiparesis and seizure	Unilateral focal stenosis of ACA, interhemispheric hematoma, subarachnoid and intraventricular hemorrhage, hydrocephalus	Focal cerebral arteriopathy post-VZV, CSF VZV DNA − and IgG −	NR	No neurologic residual, resolution of stenosis, no recurrence of AIS
Shaffer L et al., 2003 [49]	UK	Case series	1 of 5	2 y	2 mo	Transient weakness of hemibody	Unilateral focal stenosis of MCA, infarct of internal capsule	Imaging post-VZV	NR	No neurologic residual, no recurrence of AIS
Bulder MM et al.,2013 [50]	Netherlands	Case series	3	2–3 y/2.7 y	2–7 mo/4 mo (median)	Unilateral dystonic movement, hemicorea, gait disturbance	Unilateral stenosis of ICA, MCA, ACA, infarct of BG	Focal cerebral arteriopathy post-VZV CSF VZV DNA + (1) and − (1)	Acyclovir + ASA (1), ASA (2)	No neurologic residual (3) no change in stenosis (1), resolution of arteriopathy (1), no recurrence of AIS (3)
Ganesan V et al., 1997 [51]	UK	Case series	7	8 m–6 y/4 y	1 w–4 mo/1.57 mo (median)	Hemiparesis, visual impairment, signs of encephalopathy with cardiorespiratory compromise	Unilateral stenosis of MCA (3), occlusion of MCA (1) and PCA (1), 1 moyamoya	Imaging post-VZV	ASA, warfarin (1), ECMO (1)	No neurologic residual (1) dystonia (1) hemiparesis (1) persistent stenosis (1) no progression of stenosis (1) recanalization of occlusion (1) no recurrence of AIS (2) TIA (1)
Berger et al., 2000 [52]	Switzerland	Case report	1	4 y	13 mo	Hemiparesis, aphasia	Unilateral stenosis of ICA, occlusion of MCA	VZV antigen–positive giant-cell arteritis on autopsy	ASA	Fatal
Hattori H et al., 2000 [53]	Japan	Case report	1	1.5 y	3 mo	Hemiparesis	Unilateral focal stenosis MCA, infarct of BG	Focal cerebral arteriopathy post-VZV CSF VZV DNA − and IgG +	ASA	No neurological abnormalities, recurrence 5 months after
Aydin K et al., 2006 [54]	Turkey	Case report	1	2.5 y	3 w	Hemiplegia, gait disturbance, aphasia	Ischemic zone of BG	Imaging post-VZV	NR	Mild hemiparesis
Marques P et al., 2021 [14]	Portugal	Case report	1	4 y	4 mo	Nystagmus, dysmetria, ataxic gait, headache, vomiting	Focal stenosis basilar artery extending to antero-inferior cerebellar arteries bilaterally, right cerebellar infarct	Focal cerebral arteriopathy post-VZV CSF VZV DNA +	Acyclovir+ corticosteroid+ ASA	No neurologic residual, improved arteriopathy
Darteyre S et al., 2014 [55]	France	Case report	1	10 y	5 mo	Somnolence, headache, vomiting	Unilateral focal stenosis of supraclinoid ICA, occlusion of MCA	Focal cerebral arteriopathy post-VZV CSF VZV DNA +	ASA	No neurologic residual, improved occlusion, no recurrence of AIS
Bartolini L et al., 2011 [56]	Italy	Case series	4	2.2 mo–5.1 y/3.8 y	0.5–7 mo/2.9 mo (median)	Hemiparesis, hemichorea, aphasia	Unilateral stenosis of MCA, ACA and ICA (1), MCA (2) bilateral ACA, MCA (1), infarct of BG (1)	Focal cerebral arteriopathy post-VZV CSF VZV DNA + (1)	Acyclovir+ ASA+ LMWH (1), ASA (2), LMWH + ASA (1)	No neurologic residual (3) minimal hemiparesis (1), no recurrence of AIS
Daugherty W.P. et al.,2009 [57]	USA	Case report	1	14 y	NR	Headache, decreased sensation of right face, arm and hemithorax	Fusiform aneurysms of basilar artery extending to PCA, unilateral aneurysms of ICA, unilateral infarct of thalamus	Imaging post VZV CSF VZV DNA +	Acyclovir+ ASA	Stability of aneurysm, no recurrence of AIS
Beleza P. et al., 2007 [5]	Portugal	Case report	1	3 y	5 mo	Hemiparesis	Unilateral focal stenosis MCA	Focal cerebral arteriopathy post-VZV CSF VZV DNA -	Acyclovir+ ASA+ LMWH	Resolution of stenosis, no recurrence of AIS
Losurdo G. et al., 2006 [58]	Italy	Case series	4	0.5–6 y/4.1 y	2 d–1 mo/12.7 d	Hemiparesis, meningeal syndrome	Unilateral focal stenosis MCA, occlusion of MCA, infarction in the territory of MCA and of BG	Imaging post-VZV	Acyclovir + ASA (4) and LMWH (1) or Rt-PA (1)	Clinical improvement (2), not completely recovered (2) recurrence of hemiparesis and seizures (1) no recurrence (1)
Sébire G. et al., 1999 [59]	France	Case-control study	7 of 11	1 mo–15 y	9 d–9 mo/6 w	NR	NR	Imaging post-VZV	NR	Regression of arteriopathy (9) stabilization (2) [not specified for VZV group]
Nagel MA et al., 2008 [12]	Multi-national	Case series	7 of 30	1–18 y/5.4 y	6 d–20 mo/5.3 mo	NR	Focal vascular lesion: (5) mixed (2) small vessel	Focal cerebral arteriopathy post-VZV, CSF VZV DNA + (2) and IgG + (5)	Acyclovir + corticosteroid (3) Acyclovir (2)	Clinical improvement (5), stabilization (1), slow improvement (1)
Our case report	Italy	Case report	1	1 y	5 mo	Hemiparesis	Unilateral focal stenosis of MCA	Focal cerebral arteriopathy post-VZV, CSF VZV DNA +	Acyclovir + Corticosteroid + ASA	No neurologic residual, no recurrence of AIS

y: years; mo: months; d: days; NR: not reported; CSF: cerebrospinal fluid; MCA: middle cerebral artery; ICA: internal carotid artery; ACA: anterior cerebral artery; PCA: posterior cerebral artery; BG: basal ganglia; ASA: acetylsalicylic acid; LMWH: low-molecular-weight heparin; Rt-PA: recombinant tissue plasminogen activator; ECMO: extracorporeal membrane oxygenation; AIS: arterial ischemic stroke; PVA: post-varicella arteriopathy; TCA: transient cerebral arteriopathy; TIA: transient ischemic attack.

## Data Availability

The original contributions presented in this study are included in the article. Further inquiries can be directed to the corresponding author.

## Supplementary Materials

The following supporting information can be downloaded at: https://www.mdpi.com/article/10.3390/brainsci15121333/s1, Figure S1: PRISMA 2020 checklist [21].

## Abbreviations

The following abbreviations are used in this manuscript:

VZV	Varicella-Zoster Virus
WHO	World Health Organization
AIS	Arterial Ischemic Stroke
TIA	Transient Ischemic Attack
PVA	Post Varicella Arteriopathy
MCA	Middle Cerebral Artery
ACA	Anterior Cerebral Artery
ICA	Internal Carotid Artery
TCA	Transient Cerebral Arteriopathy
CSF	Cerebrospinal Fluid
v-EEG	Video-Electroencephalography
MRI	Magnetic Resonance Imaging
MRA	Magnetic Resonance Angiography
PVCA	Post Varicella Cerebral Arteriopathy
MTHFR	Methylenetetrahydrofolate Reductase
IV	Intravenous
IM	Intramuscular
ASA	Acetylsalicylic Acid
LMWH	Low-Molecular-Weight Heparin
Rt-PA	Recombinant Tissue Plasminogen Activator
